# Altered gut microbiota and metabolites in children with non-organic anorexia: a multi-omics integration study

**DOI:** 10.1038/s41598-026-52084-8

**Published:** 2026-05-11

**Authors:** Zonglong Li, Qiong Zhang, Jin Yang, Rui Lei, Wei Lu

**Affiliations:** 1 Department of Pediatric Critical Care, Shanghai Children’s Medical Center Guizhou Hospital, Shanghai Jiao Tong University School of Medicine, Guiyang, 550000 Guizhou China; 2 Department of Pediatrics, Affiliated Hospital of Zunyi Medical University/Guizhou Children’s Hospital, Zunyi, 563099 Guizhou China; 3 The First People’s Hospital of Qingzhen City, Guiyang, 551400 Guizhou China

**Keywords:** Non-organic anorexia, Children, Gut microbiota, Metagenomics, Metabolomics, Riboflavin metabolism, Gastroenterology, Microbiology

## Abstract

Gut microbiota alterations have been linked to childhood eating disorders, but the functional and metabolic changes in non-organic anorexia (NOA) remain poorly understood. This study aimed to characterize the gut microbial composition, function, and metabolic profiles in children with NOA using an integrated multi-omics approach. A case–control study was conducted involving 88 children aged 1–5 years (48 NOA, 40 healthy controls). Gut microbiota composition was assessed via 16S rRNA gene sequencing of all fecal samples. Subsequently, the five most representative samples from each group were selected for deep shotgun metagenomic sequencing and liquid chromatography-mass spectrometry (LC–MS) based non-targeted metabolomics. NOA children showed significantly higher microbial richness and diversity (Chao1, Shannon; *P* < 0.001). The NOA group had elevated Firmicutes, Bacteroidota, Bacteroides, Faecalibacterium, Subdoligranulum, and Roseburia, but reduced Actobacteriota, Bifidobacterium, and Enterococcus. Metagenomics revealed downregulated riboflavin metabolism and upregulated fat digestion/absorption pathways in NOA (*P* < 0.05). Metabolomics identified 26 differential fecal metabolites, including decreased L-carnitine derivatives and elevated tyramine glucuronide involved in bile secretion. These metabolites were significantly correlated with altered bacterial genera. Our integrated multi-omics analysis demonstrates that NOA in children is associated with a specific gut ecosystem characterized by altered microbiota structure, perturbed microbial metabolic functions (particularly riboflavin metabolism), and corresponding host-microbiota co-metabolic disturbances. These findings provide novel evidence for the disrupted "microbiota-metabolite" axis in NOA, offering new mechanistic insights.

**Supplementary Information:**

The online version contains supplementary material available at 10.1038/s41598-026-52084-8.

## Introduction

Non-organic anorexia in children, distinct from the psychiatric condition of anorexia nervosa, is a prevalent nutritional disorder characterized by persistent loss of appetite, reduced food intake, and feeding resistance without an underlying organic disease. It predominantly affects preschool children, with a high incidence around the age of six, leading to significant clinical concerns including malnutrition, growth faltering, impaired immunity, and long-term developmental consequences^1–6^. Despite its high prevalence and substantial burden on individuals, families, and healthcare systems, the precise etiology of non-organic anorexia remains elusive. Current hypotheses implicate dysregulation of the appetite control centers, altered digestive enzyme activity, and notably, disturbances within the gut microbiota^7,8^.

The gut microbiota plays a pivotal role in host physiology, functioning as a biological barrier, modulating energy harvest and metabolism, influencing endocrine signaling, and orchestrating immune system maturation^9–12^. Critically, the bidirectional communication along the microbiota-gut-brain axis is increasingly recognized as a fundamental regulator of feeding behavior and energy homeostasis^13^. Preliminary studies have consistently reported an altered gut microbial ecology in children with NOA, such as a decreased abundance of Bifidobacterium and an increased proportion of Bacteroides ^14–16^. However, these investigations, often reliant on traditional culture-dependent methods or focused solely on taxonomic composition, provide a limited view. They fail to elucidate the functional consequences of this dysbiosis and its downstream metabolic implications, leaving a critical gap in understanding the mechanistic link between gut microbes and the pathophysiology of appetite loss.

Modern high-throughput sequencing of the 16S rRNA gene has become the gold standard for profiling microbial communities, overcoming the limitations of culturability and offering a comprehensive view of bacterial diversity^17–22^. Advancing beyond taxonomy, metagenomics allows for the direct analysis of the collective genetic material (metagenome) of all microorganisms in a sample, revealing their functional potential^23–26^. Meanwhile, metabolomics, the systematic study of small-molecule metabolites, reflects the ultimate biochemical phenotype resulting from genomic, transcriptomic, and proteomic activity, offering the closest readout of an organism’s physiological state^27–29^. The integration of these multi-omics approaches—linking microbial identity (16S), functional capacity (metagenomics), and biochemical activity (metabolomics)—represents a powerful strategy to move from correlation to mechanistic insight, as demonstrated in studies of other complex disorders^30^.

To date, the functional alterations in the gut microbiome and the associated metabolic perturbations remain largely unexplored. Therefore, this study aims to bridge this knowledge gap by employing 16S rRNA gene sequencing, shotgun metagenomics, and liquid chromatography-mass spectrometry (LC–MS) based metabolomics to perform a comparative analysis of fecal samples from children with NOA and healthy controls. We seek to define the distinguishing features at the level of microbial composition, gene function, and metabolic profiles. This comprehensive approach is expected to provide novel insights into the functional mechanisms by which gut microbiota may contribute to NOA, thereby informing the future development of microbiota-targeted diagnostic or therapeutic strategies.

## Materials and methods

This study was conducted at the Pediatric Outpatient Clinic of the Affiliated Hospital of Zunyi Medical University between December 2021 and December 2022. Children aged 1 to 5 years who were diagnosed with NOA were enrolled in the Anorexia group (*n* = 48). Concurrently, 40 age-matched healthy children (Normal group, *n* = 40) undergoing routine physical examinations during the same period were randomly selected as controls. All participants were permanent residents of the local area, ensuring generally similar living and dietary environments.

Inclusion criteria: (1) For the Anorexia group: Diagnosis of non-organic anorexia according to established clinical criteria^31^, including: (a) persistent loss of appetite with a reduction in food intake by more than one-third to one-half compared to pre-morbid levels; (b) duration of symptoms exceeding one month; (c) slowed or stagnant weight gain; (d) history of improper feeding practices or poor eating habits; (e) mealtime duration ≥ 39 min. No use of antibiotics within one week prior to enrollment. Aged between 1 and 5 years. (2) For the Normal group: Healthy children aged 1 to 5 years with normal physical examination and laboratory findings. No use of antibiotics within one week prior to enrollment. (Although a one-week exclusion period may not fully reverse antibiotic-induced microbiota changes, it was chosen as a pragmatic compromise to balance recruitment feasibility; any residual effect would likely bias results toward the null, making our findings more conservative.)

Exclusion criteria: Children were excluded if they fell outside the specified age range; had current respiratory or gastrointestinal infections; had organic diseases or genetic metabolic disorders; or were unable to cooperate with the study procedures.

Specimen collection: Fresh fecal samples (5–10 g) were collected from each participant into standard sterile centrifuge tubes and immediately stored at − 80 °C until further analysis.

16S rRNA Gene Amplicon Sequencing: Genomic DNA was extracted using the QIAamp DNA Stool Mini Kit (QIAGEN, Germany). The V3-V4 hypervariable region of the bacterial 16S rRNA gene was amplified with barcoded primers on a GeneAmp® 9700 PCR System (Applied Biosystems, USA) using TransStart FastPfu DNA Polymerase (TransGen Biotech, China). The V3-V4 region was amplified using primers 338F (5′-ACTCCTACGGGAGGCAGCA-3′) and 806R (5′-GGACTACHVGGGTWTCTAAT-3′).The purified amplicons were used to construct sequencing libraries with the TruSeqTM DNA Sample Prep Kit. Paired-end sequencing (2 × 300 bp) was performed on the Illumina MiSeq platform (Majorbio, China).

Metagenomic Sequencing: Total genomic DNA was extracted using the E.Z.N.A.^®^ Soil DNA Kit (Omega Bio-tek, USA). After quality assessment, DNA was fragmented to ~ 400 bp. Sequencing libraries were prepared using the NEXTFLEX Rapid DNA-Seq Kit (Bioo Scientific, USA) and sequenced on an Illumina NovaSeq/Hiseq Xten platform (Illumina, USA).

LC–MS-based Metabolomics: Fecal metabolites were extracted from 50 mg samples using 400 μL of methanol–water (4:1, v/v) containing 0.02 mg/mL L-2-chlorophenylalanine as an internal standard, followed by homogenization and sonication. Metabolite separation was performed on a SCIEX UPLC-TripleTOF system equipped with a BEH C18 column, using a water-acetonitrile/isopropanol gradient (both with 0.1% formic acid). Mass spectrometry data were acquired in both positive and negative ion modes. Raw data were preprocessed using Progenesis QI software. Steps included: peak picking, retention time alignment, peak filtering (signal-to-noise ratio > 5), internal standard normalization (L-2-chlorophenylalanine), and removal of peaks with > 50% missing values across samples. No batch effect correction was applied as all samples were processed in a single batch.Metabolite identification was performed by matching against the HMDB and KEGG databases, and metabolite annotation confidence was assessed following the Schymanski criteria.

Bioinformatic and Statistical Analysis: (1) 16S rRNA Data:High-quality sequences were clustered into operational taxonomic units (OTUs) at 97% similarity using the SILVA database for taxonomic assignment. Alpha diversity (Shannon, Chao) and beta diversity (Bray–Curtis distance) were calculated. Differential taxa were identified using the LEfSe method (LDA score > 4, *P* < 0.05). (2) Metagenomic Data: Raw reads were quality-controlled (Fastp) and host-derived sequences were removed (BWA). De novo assembly was performed using MEGAHIT, followed by gene prediction and redundancy removal (CD-HIT) to construct a non-redundant gene catalog. Functional annotation was performed against the NR, COG, KEGG, and CAZy databases using Diamond and HMMER. (3) Metabolomic Data:Significantly altered metabolites were identified based on a Variable Importance in Projection (VIP) score > 2.5 from the OPLS-DA model and a *P*-value < 0.05 from Student’s t-test. Benjamini–Hochberg FDR correction for both metabolomic differential analysis and Spearman correlation analysis.Enriched metabolic pathways were analyzed via KEGG.

Statistical Analysis: Clinical data were analyzed using SPSS 18.0. Continuous variables are presented as mean ± SD and compared using Student’s *t* test; categorical variables are presented as counts (%) and compared using the chi-square test. A *P* value < 0.05 was considered statistically significant.

Ethics Approval and informed consent from the legal guardians of children: The study was conducted in accordance with the Declaration of Helsinki, and was approved by the Medical Ethics Committee of the AffiliatedHospital of Zunyi Medical University (Approval No. KLL-2021-299). All participants were minors (< 18 years old), and written informed consent was obtained from the parents or legal guardians of each participant prior to enrollment (and/or tissue sample collection, if applicable). The consent forms included comprehensive information about the study purpose, procedures, potential risks, benefits, and data privacy protection. All guardians voluntarily signed the forms after fully understanding the study details and had the right to withdraw at any time without prejudice.

## Results

### Clinical characteristics

A total of 88 children were enrolled in this study, including 48 in the Anorexia group and 40 in the Normal group. There were no significant differences in gender distribution or age between the two groups. However, the body mass index of children in the Anorexia group was significantly lower than that in the Normal group (Table 1).

**Table.1:** The clinical data of the two groups were compared.

Characteristic	Anorexia Group (*n* = 48)	Normal Group (*n* = 40)	Statistical test	*P* value
Gender [N(%)]			1.105	0.392
Male	21 (43.8)	22 (55.0)		
Female	27 (56.2)	18 (45.0)		
Age (years), mean ± SD	3.99 ± 1.38	3.73 ± 0.92	− 1.003	0.319
BMI (Kg/m^2^), mean ± SD	14.73 ± 1.18	16.10 ± 1.80	− 4.285	< 0.001

### Composition and diversity of the normal and anorexia groups determined by 16S rRNA gene sequencing

**Fig. 1:**
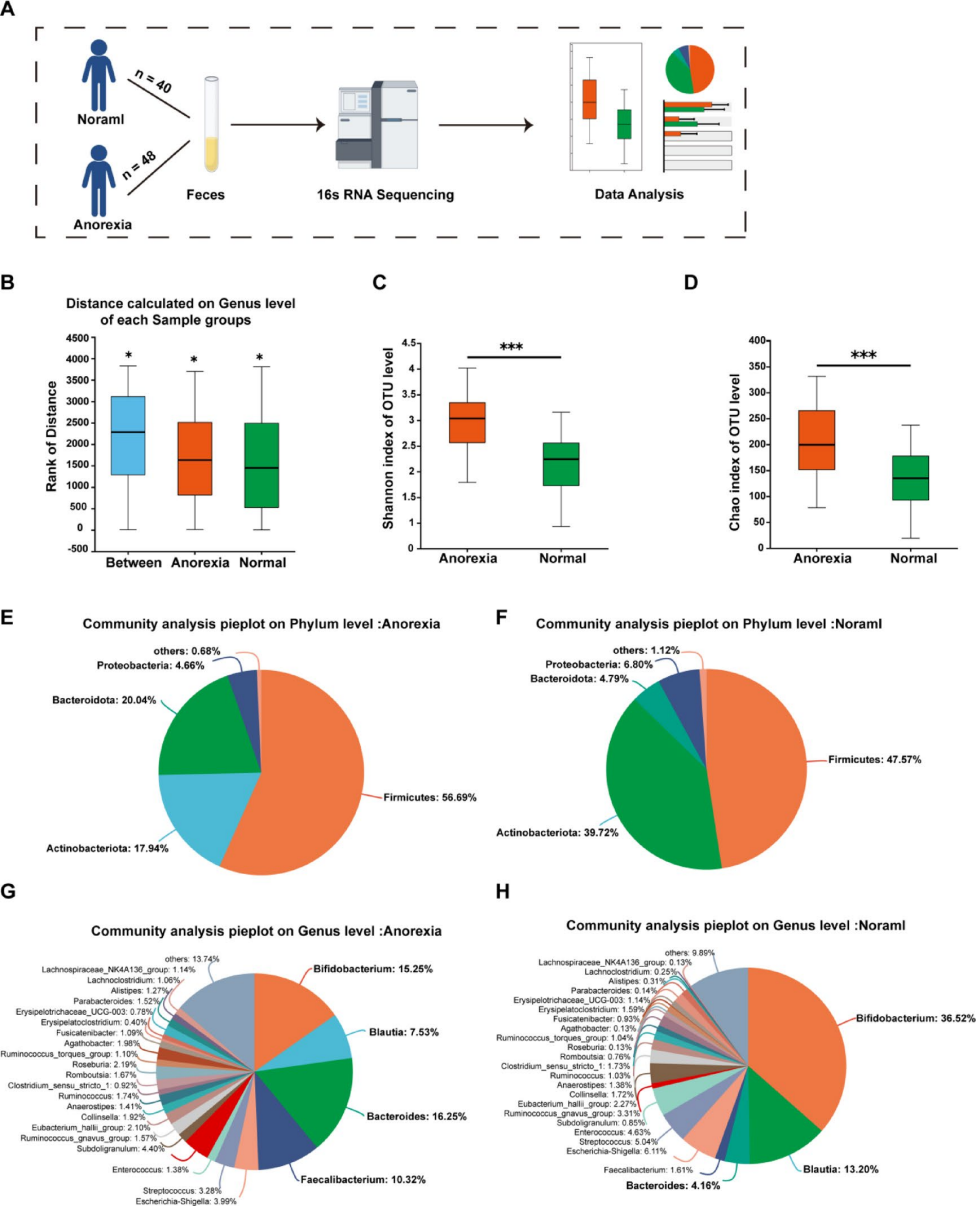
16S rRNA gene sequencing-based analysis of gut microbiota composition in normal and anorexia groups. (**A**) Sample processing schematic diagram; (**B**) analysis of similarity (ANOSIM) based on Bray–Curtis distance, showing that inter-group differences (between) are greater than intra-group variations $\mathrm{R}=0.2746$; (**C**) alpha diversity analysis:shannon index box plot; (**D**) alpha diversity analysis:chao index box chart; (**E**) Composition of dominant intestinal flora at the phylum level of the Anorexia group(with a mean relative abundance greater than 1%); (**F**) Composition of dominant intestinal flora at the phylum level of the Noraml group(with a mean relative abundance greater than 1%); (**G**) composition of dominant intestinal flora at the genus level of the Anorexia group(with a mean relative abundance greater than 1%); (**H**) composition of dominant intestinal flora at the genus level of the Noraml group (with a mean relative abundance greater than 1%).*P < 0.05,***P < 0.001.

Following high-throughput sequencing of fecal samples *n* = 88 (Fig. 1A), clustering analysis at the OTU level was performed after rarefaction based on the minimum sequence count. A total of 1,068 species were taxonomically classified across all samples, encompassing 15 phyla, 23 classes, 55 orders, 106 families, 285 genera, and 584 species.

Similarity analysis between the Anorexia and Normal groups yielded an R-value of 0.2746 (*P* < 0.01), indicating that inter-group differences exceeded intra-group variations (Fig. 1B). Comparison of alpha-diversity indices revealed that both the Chao1 and Shannon indices were significantly higher in the Anorexia group than in the Normal group (*P* < 0.001), suggesting greater microbial community richness and diversity in the Anorexia group (Fig. 1C, D).

At the phylum level, Firmicutes was the most abundant phylum in the Anorexia group, followed by Bacteroidota, Actinobacteriota, and Proteobacteria, accounting for 56.69%, 17.94%, 20.04%, and 4.66% of the community, respectively. Similarly, in the Normal group, Firmicutes was the most abundant, followed by Actinobacteriota, Proteobacteria, and Bacteroidota, constituting 47.57%, 39.72%, 6.80%, and 4.79%, respectively. Although the dominant phyla were identical between the two groups, their relative abundances differed significantly (*P* < 0.05) (Fig. 1E, F).

**Fig. 2:**
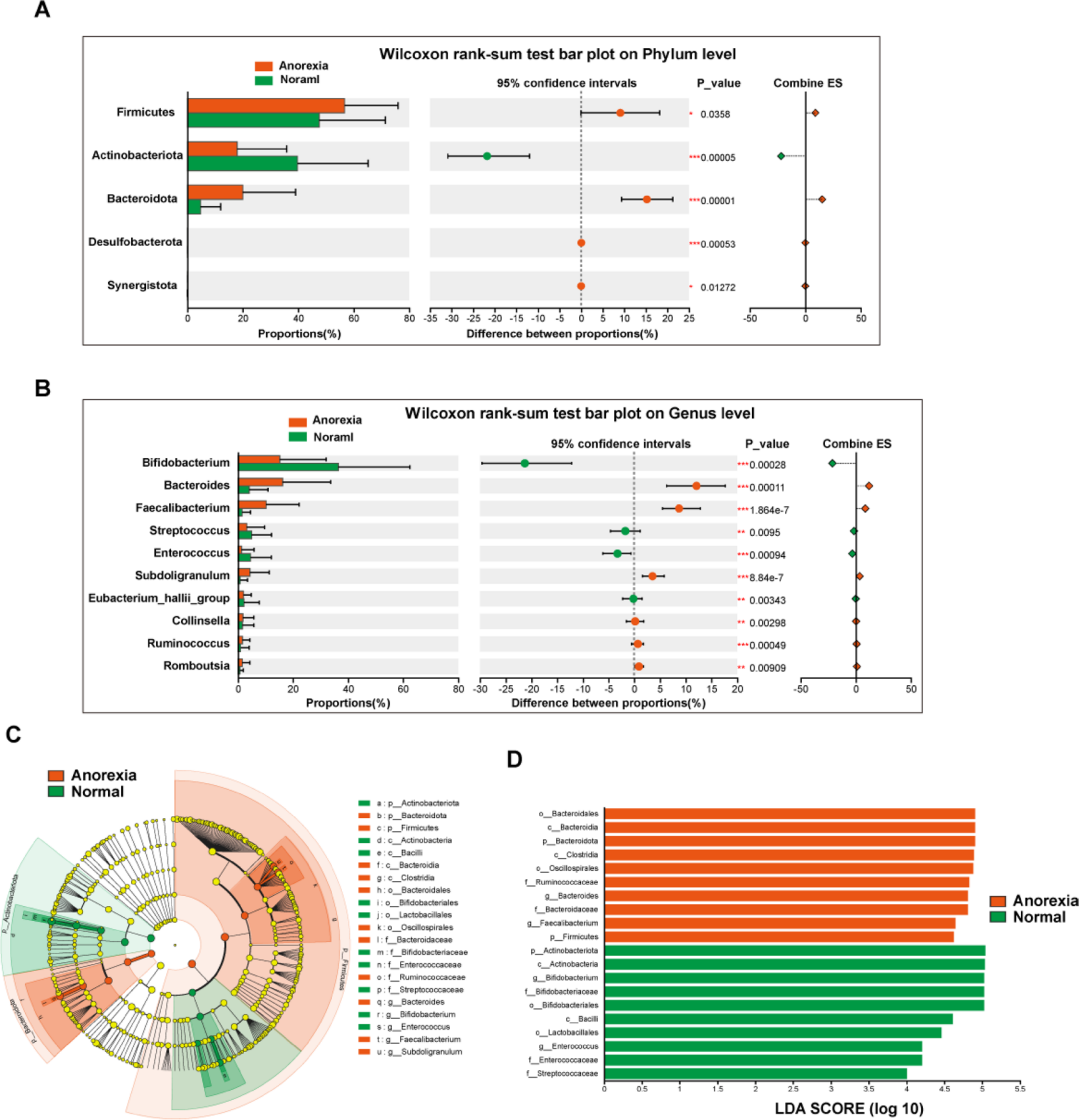
16S rRNA gene sequencing-based differential analysis of gut microbiota between normal and anorexia groups. (**A**) Differential analysis of the dominant bacterial groups at the phylum level between Anorexia and Normal groups(*P* < 0.05); (**B**) differential analysis of the dominant bacterial groups at the genus level between Anorexia and Normal groups(*P* < 0.05); (**C**) evolutionary branching diagram of Anorexia group and Normal group,from the inside out, the circles represent taxonomic ranks from phylum to genus; each dot denotes microbial taxa with an LDA score > 4.0.; (**D**) graph of LDA score results in Anorexia group and Normal group,displays only taxa with LDA score > 4 and *P* < 0.05 by Wilcoxon rank-sum test.

At the genus level, the top five most abundant genera in both groups were Bifidobacterium, Blautia, Bacteroides, Faecalibacterium, and Escherichia-Shigella. However, the composition and relative abundances of dominant genera (average abundance > 1%) differed significantly between the groups (*P* < 0.05) (Fig. 1G, H). Notably, dominant genera such as Clostridium_sensu_stricto_1, Erysipelatoclostridium, and Erysipelotrichaceae_UCG-003 were present in the Normal group but absent from the Anorexia group. Conversely, genera including Subdoligranulum, Romboutsia, and Roseburia were dominant in the Anorexia group but not in the Normal group (Fig. 2A, B).

Linear discriminant analysis Effect Size identified taxa that were significantly enriched in each group (LDA score > 4, *P* < 0.05). In the Anorexia group, the phyla Bacteroidota and Firmicutes, along with the genera Bacteroides, Faecalibacterium, Subdoligranulum, and Roseburia, were significantly enriched. In contrast, the Normal group showed significant enrichment of the phylum Actinobacteriota and the genera Bifidobacterium and Enterococcus (Fig. 2C, D).

### Microbial composition determined by metagenomic sequencing

**Fig. 3:**
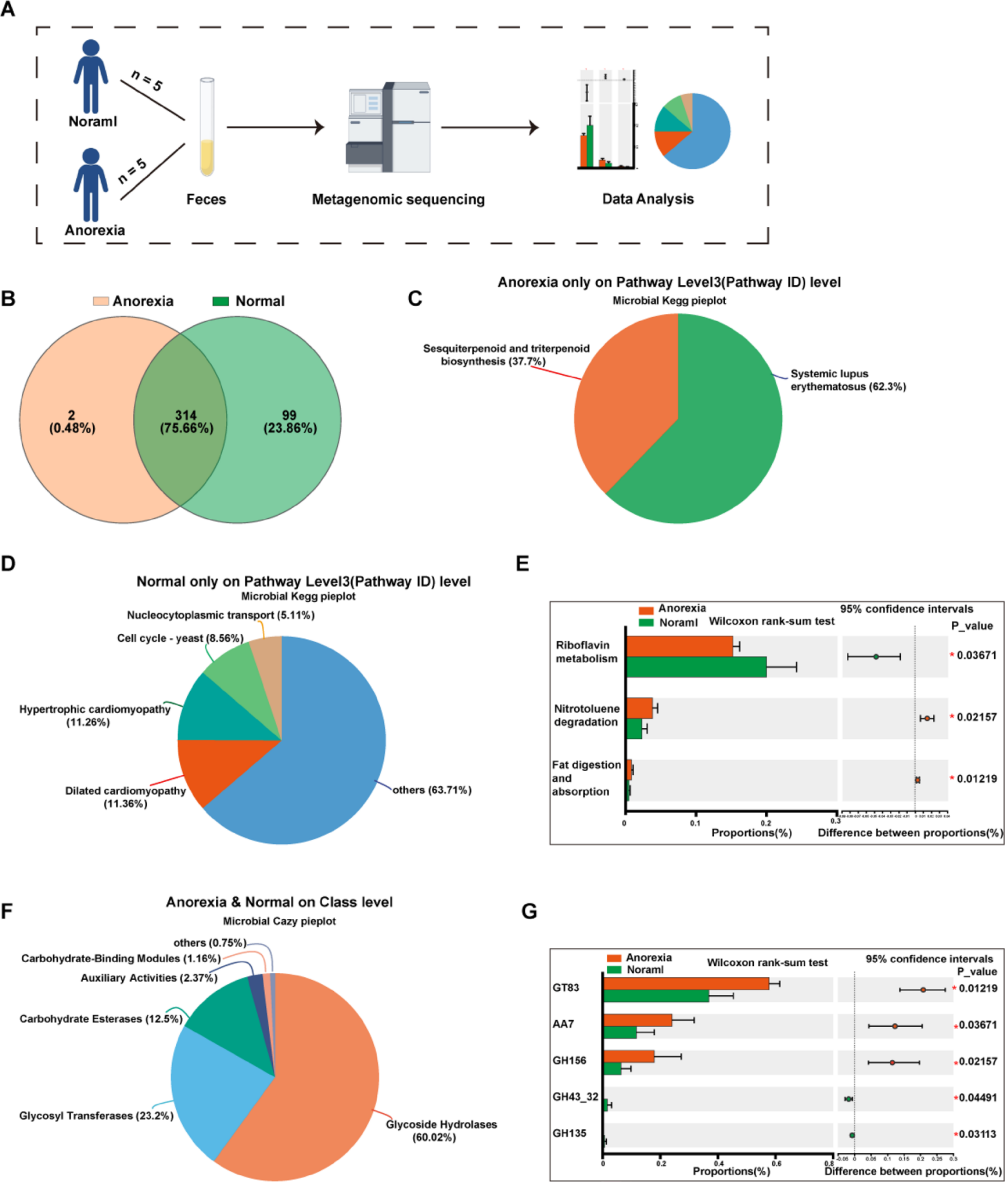
Metagenomic sequencing-based analysis of microbial composition and differences. (**A**) Sample processing schematic diagram; (**B**) number of shared and differential genes between the anorexia group and the normal group; (**C**) metabolic pathways annotated at level 3 for non-redundant genes in the anorexia group against the KEGG database.,The original pathway map is from KEGG (https://www.kegg.jp/), and modifications were performed in compliance with KEGG copyright permission. Citation:^32,33^; (**D**) Metabolic pathways annotated at level 3 for non-redundant genes in the Normal group against the KEGG database,The original pathway map is from KEGG (https://www.kegg.jp/), and modifications were performed in compliance with KEGG copyright permission. Citation:^32,33^; (**E**) Differential analysis of metabolic pathways at level 3 based on non-redundant genes annotated against the KEGG database in the anorexia and normal groups. The original pathway map is from KEGG (https://www.kegg.jp/), and modifications were performed in compliance with KEGG copyright permission. Citation:^32,33^; (**F**) Carbohydrate-active enzymes at the class level based on non-redundant genes annotated against the CAZy database in the anorexia and normal groups; (**G**) Differential analysis of carbohydrate-active enzymes (CAZymes) at the family level by aligning non-redundant genes from the anorexia and normal groups against the CAZy database.

Based on the diversity analysis data, the five most representative samples from each group were selected for shotgun metagenomic sequencing (Fig. 3A). After processing the raw sequences, the total sequences obtained accounted for over 72% of the original reads for each sample, with an average of 36,723,690 sequences per sample (range: 31,564,992 to 50,815,210). The average total sequence length per sample was 5,989,560,422 bp (range: 4,761,811,100 to 7,666,308,130 bp).

Comparative analysis of metabolites between the two sample groups identified differential metabolites(Fig. 3B).Following comparison of the non-redundant gene set with the Kyoto Encyclopedia of Genes and Genomes database and merging pathways with abundances below 5% at level 3, the metabolic pathways in the Anorexia group were found to be concentrated in sesquiterpenoid and triterpenoid biosynthesis and systemic lupus erythematosus (Fig. 3C). In the Normal group, pathways were concentrated in dilated cardiomyopathy, hypertrophic cardiomyopathy, yeast cell cycle, and nucleocytoplasmic transport (Fig. 3D). Comparative analysis revealed that genes in the Anorexia group were significantly upregulated in the "Fat digestion and absorption" and “Nitrotoluene degradation” pathways (*P* < 0.05), while being significantly downregulated in the “Riboflavin metabolism” pathway (*P* < 0.05) (Fig. 3E).

Comparison with the Carbohydrate-Active enZYmes database showed no significant difference in the categories of carbohydrate-active enzymes annotated at the class level between the two groups (*P* > 0.05). After merging classes with abundances below 1%, five main categories were identified, distributed as follows: Glycoside Hydrolases (60.02%) > Glycosyl Transferases (23.20%) > Carbohydrate Esterases (12.50%) > Auxiliary Activities (2.37%) > Carbohydrate-Binding Modules (1.16%) (Fig. 3F). At the family level, differential analysis revealed that GT83, AA7, and GH156 were significantly enriched in the Anorexia group (*P* < 0.05), whereas GH43_32 and GH135 were significantly enriched in the Normal group (*P* < 0.05) (Fig. 3G).

### Metabolomic profiling

The same ten samples used for metagenomic sequencing were further subjected to non-targeted metabolomic analysis. After data preprocessing to eliminate experimental and analytical errors, a total of 6,970 chromatographic peaks and 1,750 features were detected in positive ion mode.A total of 5939 mass spectral peaks and 1301 metabolites were detected in the negative ion mode. we used internal standard (L-2-chlorophenylalanine) for normalization. Only relative abundances are reported.

**Fig. 4:**
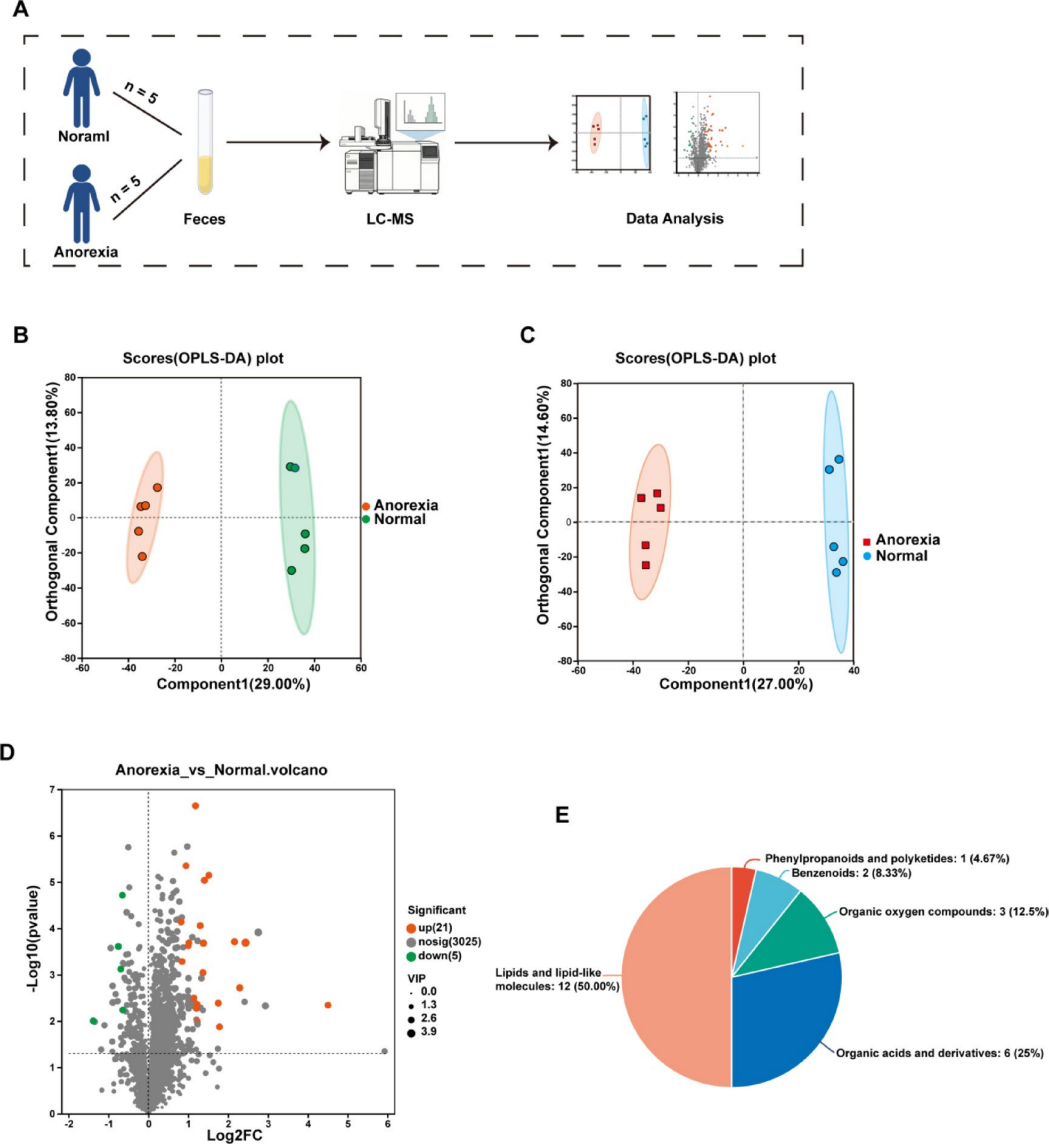
Differential analysis of differential metabolites based on metabolomic data. (**A**) Sample processing schematic diagram; (**B**) OPLS-DA score plot of anionic metabolites in the anorexia and control groups, with distinct differential metabolites identified between the two groups; (**C**) OPLS-DA score plot of cationic metabolites in the anorexia and control groups, revealing significant differential metabolites between the two groups; (**D**) Volcano plot of differential metabolites. A total of 21 metabolites were significantly upregulated and 6 were significantly downregulated in the anorexia group compared with the normal group. (**E**) HMDB superclass classification map of differential metabolites. After annotation against the HMDB database, the screened differential metabolites were classified into five major categories at the superclass level,Among the 26 differential metabolites, 24 could be classified into HMDB superclasses,the other 2 were unclassified or belonged to minor categories not shown.

Orthogonal partial least squares discriminant analysis models confirmed the presence of differential metabolites between the two groups (Fig. 4B, C). The screening criteria were variable importance in projection > 2.5, *P* < 0.05, and |log2FC|> 0.585. 21 annotated metabolites were significantly elevated, and 5 annotated metabolites were significantly reduced in the Anorexia group compared to the Normal group (Fig. 4D).

Based on the Human Metabolome Database at the superclass level, the differential metabolites were categorized into five major classes: Lipids and lipid-like molecules $50.00\mathrm{\%},12/24$, Organic acids and derivatives $25.00\mathrm{\%},6/24$, Organic oxygen compounds $12.50\mathrm{\%},3/24$, Benzenoids $8.33\mathrm{\%},2/24$, and Phenylpropanoids and polyketides $4.67\mathrm{\%},1/24$ (Fig. 4E).

**Fig. 5:**
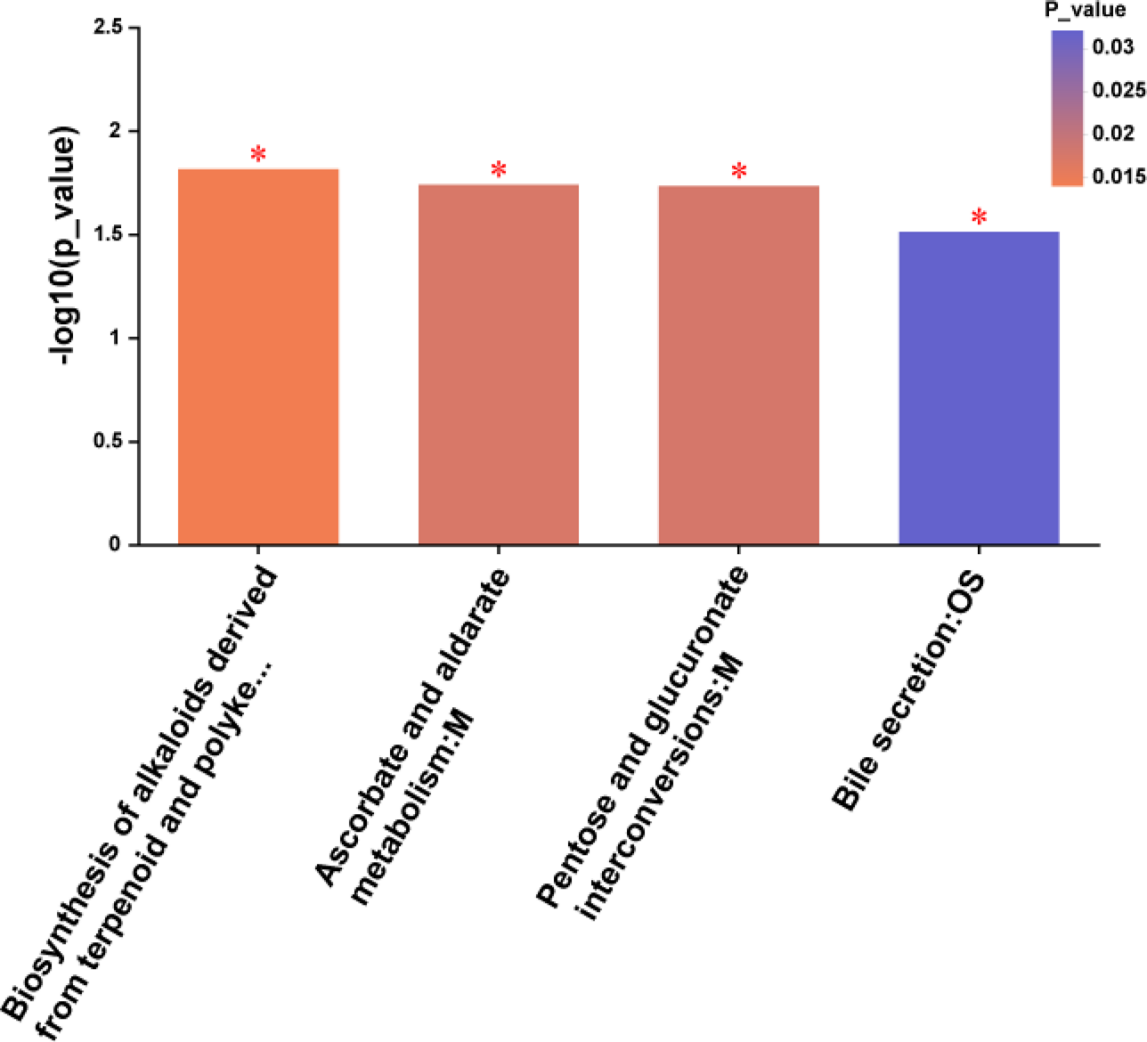
Differential metabolite pathway enrichment map,The original pathway map is from KEGG (https://www.kegg.jp/), and modifications were performed in compliance with KEGG copyright permission. Citation:^32,33^. *P < 0.05.

Metabolic pathway annotation and enrichment analysis using a hypergeometric test revealed that the differential metabolites were significantly enriched (*P* < 0.05) in four pathways: Pentose and glucuronate interconversions, Ascorbate and aldarate metabolism, Bile secretion, and Biosynthesis of terpenoids and polyketide alkaloids. The differential metabolite involved in these pathways was tyramine glucuronide (Fig. 5).

**Fig. 6:**
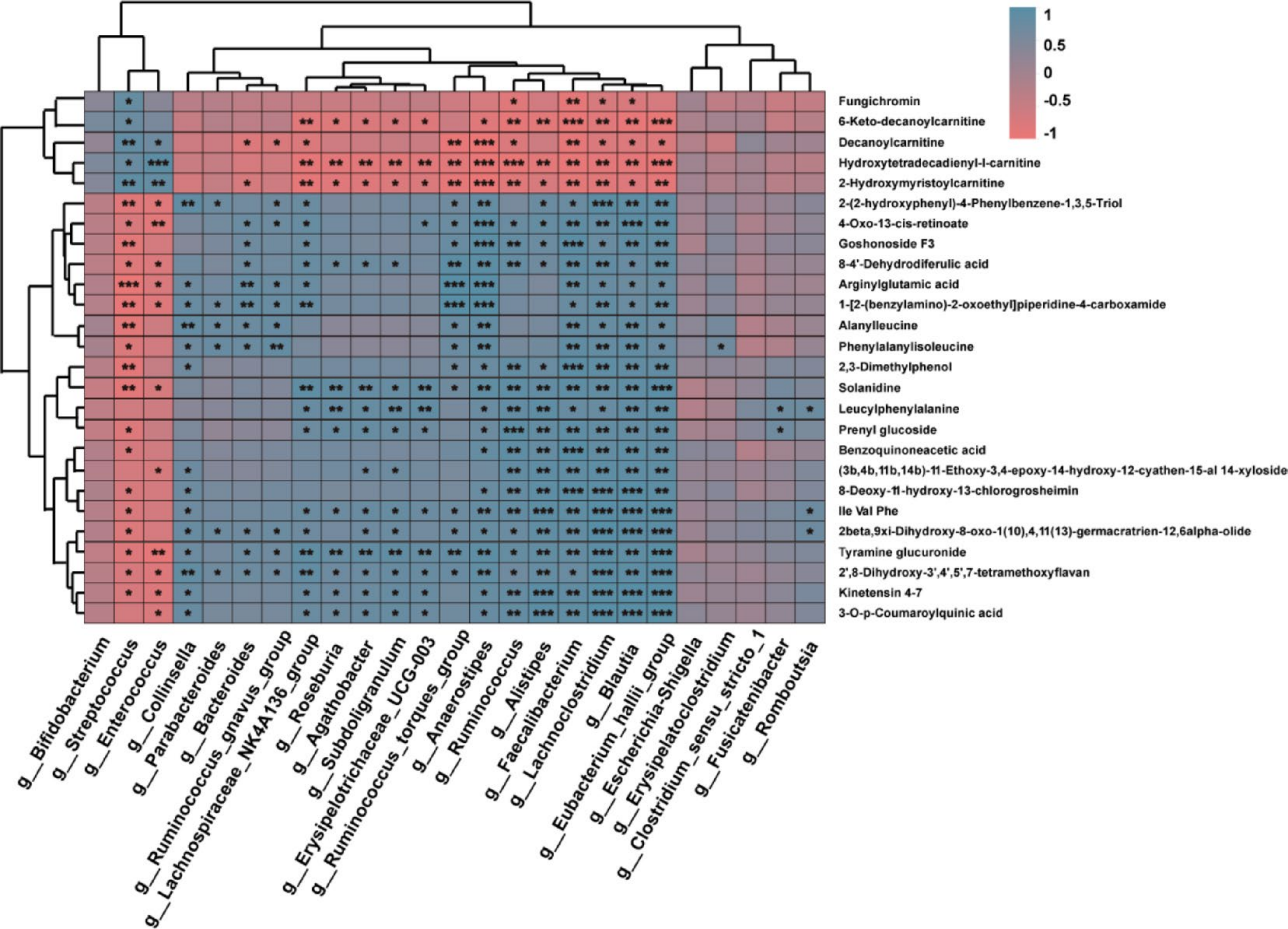
Correlation heatmap of significantly differential metabolites and dominant gut microbiota. *P < 0.05,**P < 0.01,***P < 0.001.

To elucidate the relationship between fecal metabolite changes and alterations in gut bacteria, a correlation analysis was performed between the dominant bacterial genera and the differential metabolites. As shown in Fig. 6, a significant correlation existed between gut metabolites and bacterial changes (*P* < 0.05). Metabolites significantly elevated in the Anorexia group, such as tyramine glucuronide, showed significant positive correlations with several bacteria enriched in that group, including Bacteroides, Faecalibacterium, Subdoligranulum, and Roseburia. Conversely, metabolites significantly reduced in the Anorexia group, such as 6-ketodecanoylcarnitine, showed significant positive correlations with bacteria enriched in the Normal group, such as Streptococcus and Enterococcus. No significant correlation was observed between Bifidobacterium and the differential metabolites.

## Discussion

This integrated multi omics study demonstrates that non organic anorexia, abbreviated NOA, in children is associated with significant alterations in the gut microbial ecosystem. These alterations are characterized by distinct shifts in taxonomic composition, functional potential, and fecal metabolite profiles when compared to healthy controls.

Contrary to the well-documented reduction in gut microbial diversity across multiple disease conditions^34,35^, our results demonstrated a significant elevation in alpha diversity in the NOA group. This observation highlights that the association between microbial diversity and host health is context-specific rather than following a universal linear pattern. Moreover, the one-week dietary exclusion period may also modulate gut microbial composition. The heightened microbial diversity observed in children with anorexia may stem from altered intestinal motility and prolonged gut transit time, which could promote the colonization of rare microbial taxa. Additionally, decreased nutritional intake may alleviate interbacterial competitive pressure, enabling the survival and proliferation of a broader spectrum of gut bacteria.

In line with our findings, previous studies have reported unaltered alpha diversity in multiple clinical conditions, such as breast milk jaundice and necrotizing enterocolitis, further confirming that diversity indices alone are inadequate for defining gut dysbiosis^36–39^. Instead, specific alterations in microbial community composition provide more biologically meaningful information. The NOA group displayed distinct microbial characteristics, featured by the enrichment of key taxa at the phylum and genus levels, including Bacteroidota, Firmicutes, as well as Bacteroides, Faecalibacterium, Subdoligranulum and Roseburia. Meanwhile, beneficial genera such as Bifidobacterium and opportunistic Enterococcus were markedly depleted in this cohort.

Although the NOA group presented enriched Bacteroides and an increased Bacteroidota/Firmicutes (B/F) ratio—a microbial feature widely linked to lean body phenotypes in previous literature^40–43^—we recognize that this correlation with lower BMI should not be overinterpreted in our current study. Given that individual BMI data were not collected and no correlation analysis was conducted, we avoid overstating the potential connection between the B/F ratio and body mass. Furthermore, although no classic enteric pathogens were found to be enriched in the NOA group, we cannot exclude the existence of uncultured or unannotated pathogenic taxa absent from current reference databases. Further research incorporating expanded microbial databases and culture-based approaches is required to address this limitation in future work.

The functional implications of this dysbiosis were investigated through metagenomic and metabolomic analyses. Although no statistically significant differences were found at the overall pathway level (*P* > 0.05), but the directionality (indicated by R-values) and specific pathway-level differences (e.g., riboflavin metabolism) suggest possible functional alterations that warrant further investigation. The upregulation of pathways related to fat digestion and absorption in the NOA group is congruent with the observed enrichment of short chain fatty acid, abbreviated SCFA, producing genera such as Faecalibacterium and Roseburia. SCFAs are known to stimulate the release of satiety hormones like glucagon like peptide 1, abbreviated GLP-1, which inhibits gastric emptying and reduces food intake^44^. Consequently, an enhanced capacity for SCFA production may contribute to the premature satiety and appetite loss in these patients. Conversely, the downregulation of the riboflavin metabolism pathway is particularly noteworthy. Riboflavin, also known as Vitamin B2, is essential for energy metabolism, and its deficiency can impair growth and gut function^45,46^. The concurrent reduction in Bifidobacterium, a genus harboring genes for de novo riboflavin biosynthesis in certain species^47^.The concurrent reduction in Bifidobacterium and downregulation of riboflavin metabolism is an interesting association, but causality cannot be inferred. It is possible that Bifidobacterium contributes to riboflavin availability, or that other factors drive both changes. This hypothesis requires experimental validation.

Metabolomic profiling further corroborated these functional disturbances. The significant decrease in L carnitine derivatives, such as 6 ketodecanoylcarnitine, points to altered fatty acid beta oxidation and compromised energy derivation from fat stores. Such depletion is known to be associated with metabolic disorders like insulin resistance and endocrine dysregulation^48,49^. Although the role of L carnitine in appetite regulation appears complex, its reduction may reflect a broader state of metabolic dysregulation.

More directly, the elevated level of tyramine glucuronide, a metabolite significantly correlated with enriched genera like Bacteroides, was linked to the bile secretion pathway. Tyramine, a biogenic amine produced by gut bacteria through amino acid decarboxylation, undergoes glucuronidation as a key hepatic detoxification and excretion route^50^. Bile acids are potent regulators of metabolism and appetite, influencing the secretion of anorexigenic gut hormones such as peptide YY^51^. Gut microbes, particularly Bacteroides, are known to modify bile acid composition, and their overgrowth could perturb bile acid signaling^52^. Therefore, we hypothesize that the dysbiotic gut microbiota in NOA may indirectly interfere with appetite regulating bile acid signaling by altering the levels of related metabolites, thereby playing a role within the microbiota gut brain axis.

This aligns with our Spearman correlation analysis, which revealed significant associations between specific differential metabolites and differential bacterial genera, reinforcing the concept of a disrupted microbiota metabolite axis in non organic anorexia.

This study has several inherent limitations. First, detailed dietary intake was not systematically recorded; thus, we cannot rule out the possibility that intergroup dietary discrepancies may have driven the observed shifts in gut microbiome and metabolome profiles. Future investigations should integrate comprehensive nutritional assessment to minimize such confounding factors. Second, the relatively small sample size (n = 5 per group) for metagenomic and metabolomic analyses may lead to potential selection bias and insufficient statistical power. In addition, representative sample screening was performed based on 16S rRNA sequencing data, and independent validation in a larger cohort is therefore necessary. Third, untargeted metabolomics has inherent technical limitations, and further verification using authentic reference standards is warranted.

Furthermore, the present study failed to clarify the underlying molecular mechanisms or provide orthogonal evidence to support causal inference. Accordingly, our results remain correlative and hypothesis-generating. Follow-up mechanistic studies, including in vitro experiments and animal model validation, are essential to establish the causal interplay between specific gut microbes, differential metabolites, and childhood appetite regulation.

## Conclusions

In summary, this multi omics study provides a comprehensive characterization of the gut ecosystem in children with non organic anorexia:

Altered Gut Microbiota Structure: We identified a distinct microbial signature associated with NOA, characterized by a significant increase in the relative abundances of Bacteroides, Faecalibacterium, Subdoligranulum, and Roseburia, alongside a marked decrease in Bifidobacterium. This suggests that these structural shifts in the gut microbiota may be implicated in the pathogenesis or progression of anorexia.

Downregulation of Riboflavin Metabolism:Metagenomic functional analysis revealed a significant downregulation of the riboflavin metabolism pathway in the gut microbiota of anorexic children. Given the critical role of riboflavin (Vitamin B2) in energy metabolism, we speculate that the inhibition of this pathway may contribute to the pathological process of anorexia by affecting host energy homeostasis.

Dysregulation of Specific Metabolites:Metabolomic analysis indicated a significant decrease in L carnitine derivatives and an increase in tyramine glucuronide levels in the feces of anorexic children. These metabolite changes showed significant correlations with specific gut microbiota alterations, highlighting a concomitant host microbial co metabolic disorder in anorexia, particularly within lipid metabolism and bile acid related pathways.

Collectively, our findings reveal characteristic alterations in the gut microenvironment of children with non organic anorexia at the compositional, functional, and metabolic levels. This work provides novel evidence for understanding the "microbiota metabolite appetite" regulatory mechanism and points out the direction for future microecological interventions aimed at improving childhood anorexia.Further experimental validation, including in vitro assays and animal models, is needed to clarify the causal relationships between specific gut microbiota, circulating metabolites, and appetite regulation.

## Supplementary Information

Below is the link to the electronic supplementary material.

Supplementary Material 1

Supplementary Material 2

## Data Availability

The datasets generated and/or analysed during the current study are available in the NCBI SRA repository, PRJNA1416374 (https://www.ncbi.nlm.nih.gov/bioproject/PRJNA1416374), and The metabolomics data have been deposited in Metabolomics Workbench (https://www.metabolomicsworkbench.org/) with Study ID ST004630.

## Abbreviations

CAZy: Carbohydrate-active enzymes
COG: Cluster of orthologous groups of protein
GH: Glycoside hydrolases
GLP-1: Glucagon-like peptide-1
GT: Glycosyl transfeses
HMDB: Human metabolome database
KEGG: Kyoto encyclopedia of genes and genomes
LC–MS: Liquid chromatography–mass spectrometry
LDA: Linear discriminant analysis
NOA: Non-organic anorexia
NR: Non-redundant protein sequence database
SCFA: Short-chain fatty acid
